# P-1564. The Influence of Chronic HIV and Obesity on Post-COVID Immune Activation and Clinical Manifestations

**DOI:** 10.1093/ofid/ofaf695.1744

**Published:** 2026-01-11

**Authors:** Skye Opsteen, Mildred Perez, Carson Norwood, Aoyjai Montgomery, Emily Levitan, Nathan Erdmann

**Affiliations:** University of Alabama at Birmingham, Birmingham, AL; University of Alabama at Birmingham, Birmingham, AL; University of Alabama at Birmingham, Birmingham, AL; University of Alabama at Birmingham, Birmingham, AL; University of Alabama at Birmingham, Birmingham, AL; University of Alabama at Birmingham, Birmingham, AL

## Abstract

**Background:**

People with HIV (PWH) are 2-4x more likely to develop long COVID than people without HIV (PWOH), potentially due to the heightened immune activation and inflammation typical of chronic HIV despite effective antiretroviral therapy. We evaluated how long COVID symptoms in PWH compare to PWOH, and whether the shifts in monocyte and T cell populations seen in PWH are exacerbated in long COVID.

**Figure:**
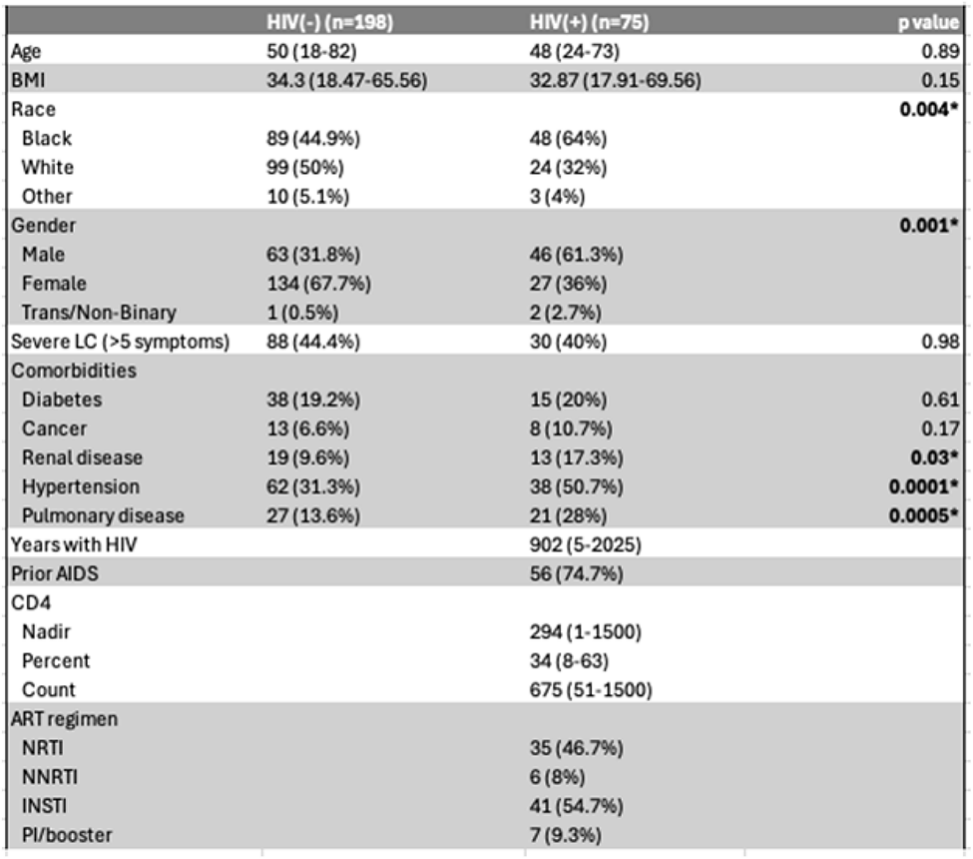
Cohort Demographics LC, long COVID; INSTI, integrase strand transfer inhibitor; NNRTI, non-nucleoside reverse transcriptase inhibitor; NRTI, nucleoside reverse transcriptase inhibitor; PI, protease inhibitor. Mann-Whitney U test or Fisher exact test performed for statistical analysis.

**Figure:**
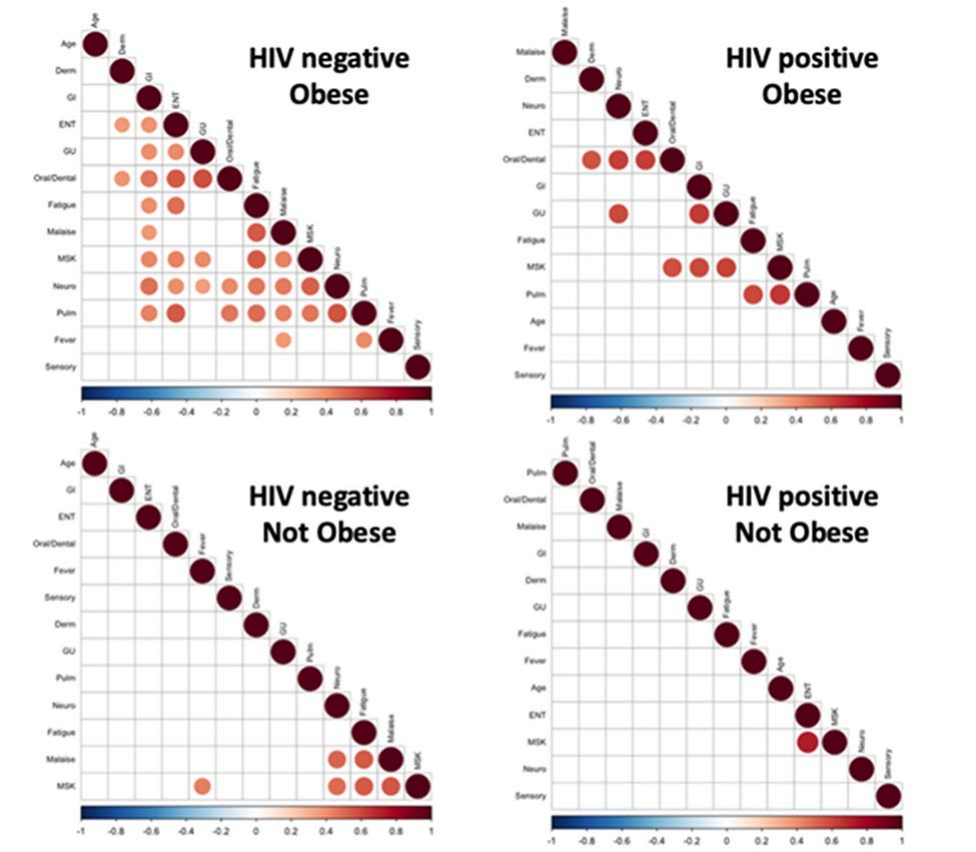
Long COVID symptom clusters are impacted by HIV and Obesity status Obesity defined as a BMI ≥ 30. Symptom clusters were determined via hierarchical clustering methods in R. Correlation values only shown if p ≤ 0.0001.

**Methods:**

We captured demographic and medical data who enrolled in our local prospective COVID-19 cohort. Participants completed a symptom survey (average 890 days [range 606-1174] after their first reported COVID-19 infection). Participants were grouped by HIV diagnosis prior to a COVID-19 infection as PWH (n=75) or PWOH (n=198). We performed flow cytometric analyses on peripheral blood mononuclear cells from PWH with (n=19) and without (n=23) long COVID and PWOH with (n=55) and without (n=66) long COVID. Cells were stained for surface markers of activation/exhaustion and monocyte subsets.

**Figure:**
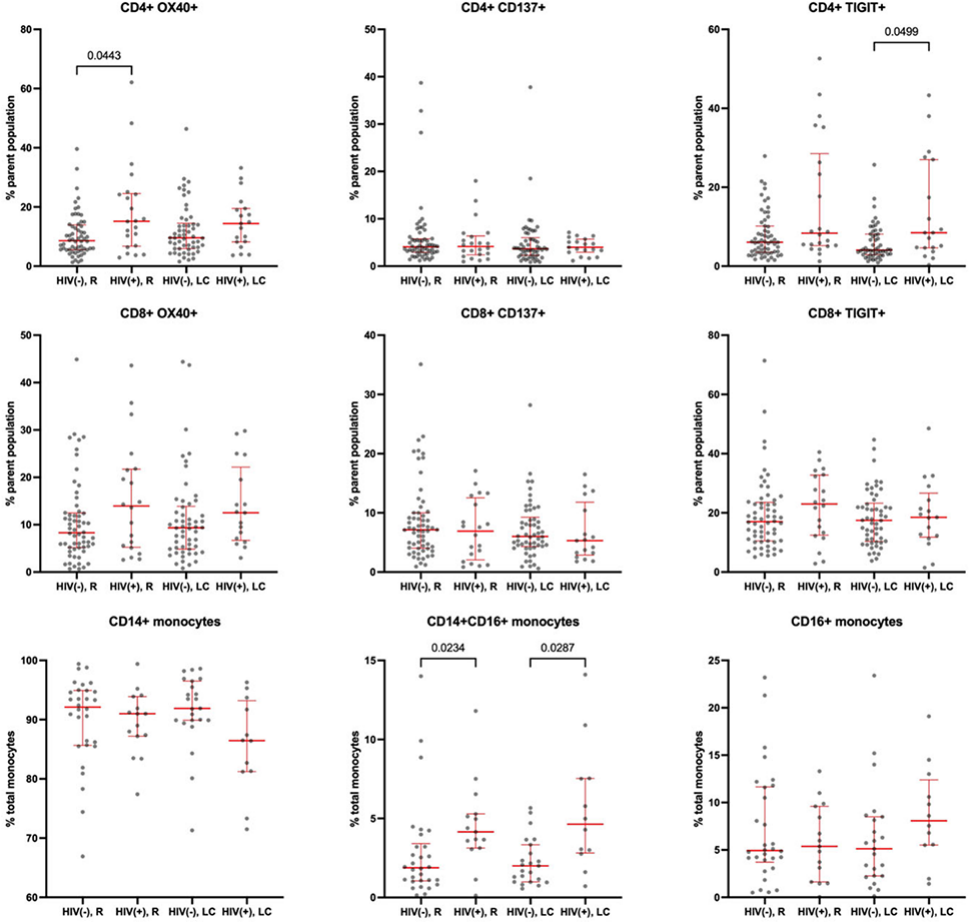
Peripheral immune responses are driven by HIV, not long COVID. R - Recovered from acute COVID-19. LC - severe long COVID (> 5 symptoms). HIV(-) - People without HIV. HIV(+) - people with HIV. Differences in T cell marker expression and monocyte subset frequencies were analyzed via Kruskal-Wallis with post-hoc Dunn's.

**Results:**

PWH reported higher frequencies of ENT symptoms as compared to PWOH (50% vs 34%). PWOH with obesity (BMI ≥ 30) reported higher frequencies of post-exertional malaise (78% vs 22%) and cardiovascular symptoms (68% vs 47%) compared to non-obese PWOH. Further, PWOH with obesity were more likely to have severe long COVID ( > 5 symptoms) (72% vs 38%). Notably, this BMI effect was not observed in PWH. Heatmaps were generated to further evaluate the impact of HIV and obesity status on symptom clustering. Correlative data among those with obesity suggests less heterogeneity in long COVID clinical presentations in PWH than PWOH (10 vs 33 highly correlated symptoms). Despite observing HIV- and obesity-related shifts in symptoms, peripheral immune responses differed by HIV-status alone.

**Conclusion:**

Our findings demonstrate that although peripheral immune response differences were HIV-specific, long COVID clinical manifestations differed by both HIV and obesity status. These insights suggest potential differences in the underlying pathogenesis of long COVID, which should be taken into the account when considering potential treatments for patients with inflammatory phenotypes of long COVID and other post-viral syndromes.

**Disclosures:**

All Authors: No reported disclosures

